# Peer support in doctoral studies: insights from qualitative surveys and interviews with PhD students

**DOI:** 10.1080/17482631.2026.2756027

**Published:** 2026-11-05

**Authors:** Anita Øgård-Repål, Monika Øgård, Tina Binfield-Skøie

**Affiliations:** a Department of Health and Nursing Science, University of Agder, Kristiansand, Norway; b Department of Education, University of Agder, Kristiansand, Norway

**Keywords:** Sense of belonging, PhD community, informal support, peer collaboration, peer support

## Abstract

**Purpose:**

This study explores how PhD students perceive peer support and how interaction with peers fosters a sense of belonging. The study addresses belonging as a fundamental psychological and relational process of how peer support is experienced and functions as a sustaining factor in doctoral education.

**Methods:**

The study adopts an exploratory qualitative design with an interpretive approach. The data consist of qualitative survey responses (*n *= 73) and semi-structured individual interviews (*n *= 14) conducted at a Norwegian university. The analysis employed an innovative, AI-assisted stepwise reflexive thematic analysis.

**Results:**

The results highlight both emotional and academic dimensions of peer support. First, informal peer arenas act as buffers against emotional strain, contributing to motivation and well-being. Second, peer collaboration fosters the development of academic skills and professional identity. Together, the results indicate that peer relationships strengthen students’ sense of belonging and function as a key motivational resource throughout the PhD journey.

**Conclusions:**

The study contributes insight into how informal, student-initiated peer support complements formal structures in doctoral education. It conceptualises belonging as a relational process enacted in everyday interactions and highlights institutional implications, emphasising the need to facilitate inclusive, flexible peer environments that support well-being, motivation, and completion.

## Introduction

The progress of PhD students is shaped by multiple factors, with mental health and well-being playing a particularly critical role (Hazell et al., 2020). PhD students face numerous challenges, including disruptions to their research activities and balancing work and domestic responsibilities. These pressures can significantly affect their mental health and well-being, and may impact their ability to complete their PhD’s within the expected timeframe (Hazell et al., 2020). While peer relationships are widely recognised as supportive, existing research often overlooks the informal, student-initiated, and non-evaluative dimensions of peer interaction. This study addresses this gap by exploring how these informal peer spaces, acting as a crucial relational infrastructure, serve not only as emotional support but as a vital conduit for academic motivation, professional identity socialisation, and social integration (Martínez-García et al., 2025; Wegener et al., 2016).

Peer support has increasingly been recognised as a mechanism that provides academic and emotional benefits where PhD students may experience a sense of belonging, a core psychological need that supports motivation, persistence, and well-being (Baumeister & Leary, 1995; Hagerty et al., 1996). While often understood as an emotional experience, belonging is also a relational process shaped through everyday interactions among peers. In doctoral education, these collegial interactions are central, and a lack of meaningful relationships may lead to experiences of exclusion and non-belonging within academic communities (Svendby, 2026). In contrast to institutionally facilitated forms of mentoring, this study examines self-initiated peer support amongst PhD students. This exploration is particularly salient within the Norwegian higher education context, where PhD students hold a dual status as university staff with employment benefits and teaching responsibilities (HK-dir, 2025; NIFU, 2019). While providing financial stability, this employment-based structure introduces complex role negotiations, making informal peer communities a vital, yet under-researched, resource for sustaining a sense of academic belonging. The aim is to explore how PhD students perceive peer support and how peers may serve as sustaining factors throughout the PhD journey. Specifically, we ask:

*How do PhD students perceive peer support during their doctoral education, and in what ways can peer interaction foster a sense of belonging throughout the PhD journey?*

It is widely recognised that PhD students are more likely than other students and the general population to experience poor mental health and well-being (Hazell et al., 2020). Recent systematic reviews confirm that burnout, acute psychological distress, and dropout intentions continue to persist at disproportionately high rates in contemporary higher education (Martínez-García et al., 2024). Studies indicate that half of PhD students experience psychological distress, with one in three at risk of common psychiatric disorders (Levecque et al., 2017). A recent population-level study from Sweden substantiates the concern that PhD students are more vulnerable to mental health and well-being issues, showing that psychiatric medication use among PhD students increases by 40% during the course of their studies, peaking in the fifth year before declining post-graduation (Bergvall et al., 2025). This trend suggests that the doctoral period itself may contribute to deteriorating mental health, underscoring the importance of relational and institutional support mechanisms, particularly for international PhD candidates, who may experience isolation due to language barriers and limited collegial integration (Svendby, 2026).

Motivational aspects often decline during the initial years of doctoral studies (Gonzalez et al., 2021). Access to supportive peer environments is recognised as a key factor in fostering motivation, well-being and academic progress. Here, peers are defined as colleagues of the same academic rank and from the same university (cf. Boud & Lee, 2005; Wegener et al., 2016). Peer support is a critical factor in the well-being of PhD students, providing both academic and emotional benefits, and its importance for successfully navigating the PhD journey (Boud & Lee, 2005; Gardner, 2010; Wegener et al., 2016). Research suggests that regular peer interactions help mitigate feelings of isolation, provide practical academic support, and foster a supportive and motivating environment. Institutions can play a key role in supporting these dynamics by facilitating opportunities for peer engagement and collaboration (Martínez-García et al., 2024, 2025). Despite its recognised value, the mechanisms through which peer support fosters belonging and sustains motivation remain underexplored, particularly in informal and student-initiated contexts.

## Conceptualisation of peer support in doctoral studies

Intellectual challenges, emotional turbulence, and social isolation often characterise the doctoral journey (Hazell et al., 2020; Levecque et al., 2017). While supervision has traditionally been emphasised, recent research highlights the critical role of peers in supporting PhD students’ well-being through both academic and emotional benefits. Peer networks thus play an important role in navigating doctoral challenges (Boud & Lee, 2005; Gardner, 2010; Wegener et al., 2016).

Recent systematic reviews on doctoral well-being confirm that mental health challenges, such as burnout, psychological distress, and dropout intentions, continue to persist in contemporary academia (Hazell et al., 2020; Mahsood et al., 2025; Martínez-García et al., 2024). This has led researchers to advocate for more comprehensive, systemic and relational support structures to safeguard PhD students' psychological health throughout their studies (Martínez-García et al., 2024).

Peer support can be understood as a relational mechanism fostering a sense of belonging; feeling accepted, valued, and emotionally connected (Baumeister & Leary, 1995; Hagerty et al., 1996). Informal interactions, such as lunches, spontaneous conversations, and writing groups, create supportive spaces that enhance emotional safety and academic engagement. However, navigating the social environment of doctoral education is highly complex, as peer dynamics are continuously negotiated alongside systemic academic pressures and demanding work organisations (Levecque et al., 2017; Martínez-García et al., 2025). While collaborative peer relationships are a vital resource, PhD students must constantly balance academic demands, research milestones, and structural stressors, which can sometimes turn collegial spaces into arenas of implicit comparison or performance anxiety (Gonzalez et al., 2021; Martínez-García et al., 2025). This highlights the need to establish supportive, informal, student-initiated peer environments that operate outside formal evaluative structures, allowing PhD students to safely share vulnerabilities and buffer the emotional strain inherent in the PhD trajectory (Martínez-García et al., 2025; Wegener et al., 2016).

Meschitti (2019) shows how peer learning within research teams occurs through both structured and informal interactions, positioning it as central to developing disciplinary knowledge and scholarly skills. Activities such as reading groups, discussions, and feedback foster intellectual risk-taking and strengthen students’ sense of agency (Meschitti, 2019).

Similarly, Wang and Min (2023) demonstrate that peer support contributes to skill development and research satisfaction, offering practical help and emotional reassurance often less accessible in supervisory relationships. Matheka et al. (2025) further show that while supervision predicts success, peer support is crucial for fostering belonging, which is linked to persistence and timely completion, particularly where institutional support is limited. This developmental trajectory can be theoretically understood through Lave and Wenger (1991) Communities of Practice (CoP) framework, which conceptualises the doctoral journey as a situated social learning process (Boud & Lee, 2005). Engaging in authentic, collaborative tasks, such as peer-review groups or joint academic problem-solving, creates ‘protected spaces’ where PhD students transition from peripheral participants to central, legitimate contributors of the academic community (Boud & Lee, 2005; Meschitti, 2019; Wegener et al., 2016). Through this relational, practice-based learning, PhD students not only co-construct essential academic skills but also experience profound development of their scholarly identity and research self-efficacy (Gonzalez et al., 2021; Meschitti, 2019).

Overall, peer support emerges not merely as a supplement to supervision, but as a distinct pedagogical and relational resource that enhances academic development, emotional resilience, and a sense of belonging within the academic community.

### To belong

Belonging can be understood as a relational and dynamic process with implications for health and well-being (Hagerty et al., 1992, 1996). According to Hagerty et al. (1992), a sense of belonging is established through two distinct, defining dimensions: (1) *valued involvement*, which represents the individual's experience of feeling valued, needed, accepted, and respected by others, and (2) *fit*, which refers to the person's perception that their personal characteristics, values, and goals articulate with or complement the surrounding system (Hagerty et al., 1992). In higher education, these dual dimensions position belonging as central to social and psychological well-being (Hagerty et al., 1992, 1996). For PhD students, both dimensions are continuously negotiated within the academic landscape: while informal, student-initiated social spaces primarily cultivate emotional safety and *valued involvement*, collaborative academic endeavours foster intellectual alignment and *fit* within the wider scholarly community (Boud & Lee, 2005; Meschitti, 2019; Wegener et al., 2016).

The need to belong is considered a fundamental human drive, as individuals seek meaningful relationships through shared experiences (Baumeister & Leary, 1995). Such interpersonal bonds function as protective resources, particularly in times of stress (Baumeister & Leary, 1995; Martínez-García et al., 2024).

In higher education, belonging is closely linked to academic success, retention, and student satisfaction (Strayhorn, 2012; van Kessel et al., 2025), and is especially important for marginalised groups facing structural barriers (Vaccaro & Newman, 2017). Supportive environments and meaningful interactions are key to fostering belonging (Amin et al., 2025). Furthermore, recent empirical research in higher education has substantiated the direct pathways between social interactions, sense of belonging, and psychological well-being (Allen et al., 2021; Caligiuri et al., 2020; Wang et al., 2015). Quantitative mediation analyses demonstrate that a sense of belonging at university serves as a critical cognitive and emotional bridge (Allen & Kern, 2017; Dost & Smith, 2023). Specifically, it has been shown to partially mediate the positive relationship between peer relationships and psychological well-being (Brandseth et al., 2019; Zhang et al., 2025), directly contributing to improvements in self-esteem, effective coping strategies, and significant reductions in anxiety, depression, and loneliness (Richard et al., 2022; Wu & Dong, 2024). In doctoral education, peer relations may enhance belonging, particularly where institutional support is limited (Matheka et al., 2025). Through shared academic and emotional experiences, students develop relational ties that affirm their place in academia and mitigate isolation (Gonzalez et al., 2021; Svendby, 2026; Wegener et al., 2016). This study argues that peer communities can foster PhD students’ sense of belonging as a motivational resource during doctoral studies.

## Methodology

This exploratory qualitative study adopted an interpretive and reflective approach to explore PhD students’ experiences of peer support. The study draws on two qualitative data sources: responses from an open-ended qualitative survey and semi-structured individual interviews conducted in 2024–2025. Although the survey included a limited number of background variables, the present study is based exclusively on qualitative data derived from open-ended survey responses and individual interviews.

This study is situated within the Norwegian higher education context, which offers a distinctive framework for exploring the well-being of PhD students. In Norway, most PhD students are employed as university staff, combining research with teaching responsibilities over a typical four-year period (HK-dir, 2025). Other students have no teaching responsibilities and have a three-year period for their doctoral studies. These positions are fully funded and include employment benefits, contrasting with models in many other countries where PhD studies are often self- or scholarship-funded (NIFU, 2019). While this employment-based structure provides financial stability and professional integration, it also introduces complex role negotiations as students balance their identities as both students and employees (NIFU, 2019).

Survey participants were recruited through email invitations extended by a PhD network at a Norwegian university in 2024. The invitations consisted of information about the study, and PhD students were asked to complete a self-administered survey electronically via an online survey platform (SurveyXact, Ramboll Management Consulting, Aarhus, Denmark). The material from the survey consists of 73 responses.

The material from the survey consists of 73 responses. The qualitative survey included open-ended questions aimed at gaining a broad understanding of PhD students’ experiences with motivation during their PhD journeys. In line with qualitative survey methodology, the survey generated textual data that were analysed alongside the interview material. Examples of the different sections were: factors of motivation (individual and collective), collaboration with supervisors, and academic and personal affiliations and collaborations. An initial pilot study was conducted with PhD students who were near completion of their theses. Conducting a pilot study aligns with recommendations for qualitative surveys (Kvale et al., 2015). This led to minor modifications before inviting all PhD students at a Norwegian university to participate.

Participants who completed the survey were invited to individual interviews by providing their contact information. Fourteen PhD students participated in the interview study. Participants were at different stages in their PhD, some at the beginning and others having recently submitted their theses. The PhD students were recruited from a range of disciplines across the University, including technology, education, health sciences and biology, to name just a few. An interview guide was developed with open-ended questions, covering topics such as managing workload, PhD processes, and motivation for pursuing doctoral work. We conducted semi-structured interviews in spring 2025, either on campus or online, depending on participants’ preference, lasting about one hour. Interviews were recorded and transcribed verbatim. The aim was to gain insight into how PhD students experience their situations and life conditions (Kvale & Brinkmann, 2015). In this study, the interviews allowed us to trace how PhD students structure their work and sustain motivation throughout the PhD process.

### Ethical considerations

The study was assessed by the Norwegian Agency for Shared Services in Education and Research and approved by the Faculty of Health and Sport Sciences Research Ethics Committee, and follows the Norwegian Guidelines for Research Ethics in the Social Sciences and the Humanities (NESH, 2021). The study was carried out in accordance with the principles of the Declaration of Helsinki. All data and participants are anonymised.

For the qualitative survey, informed consent was obtained at the outset, including contact details for the project leader (Author 1). The survey was anonymised by design. Participants interested in interviews could provide contact information in a final question; this information was deleted after the project.

Written informed consent was also obtained prior to the interviews, and participants could ask questions before and after. The author's positions as current PhD students (authors 2 and 3) or recently submitted PhD student (author 1) were an ethical awareness of acknowledging the participants as experts on their experiences and perspectives, even if these were not similar to our own. At the same time, shared experiences appeared to enhance trust, enabling participants to reflect on their PhD journeys.

AI was used as a dialogical partner in the analysis, prompting a thorough re-examination of initial interpretations. This required strong familiarity with the data, but also facilitated new insights, particularly across survey and interview material. While AI supported more nuanced exploration, the final analysis remained grounded in the researchers’ own work, as presented in this study.

### The analytical process

The analysis was inspired by thematic analysis (Braun & Clarke, 2022). This involves a reflexive approach to the material, where the aim is to gain an understanding of recurring themes across the material. We approached the analysis in three steps (illustrated in Table I). Firstly, we started with a familiarisation with the material. In this process, a juxtaposition of the qualitative surveys and the individual interviews was categorised into themes. While the surveys provide a broad overview of the PhD students' experiences of peer support, the interviews offered deeper insights into the challenges and opportunities of engaging with other PhD students. Three overarching themes occurred: peer support, supervision, and PhD students’ processes of structuring their work. Because all three themes are complex, we turn our attention to the different themes in separate studies, here focusing on *peer support*. In the analysis of the theme, peer support, we turned the attention to how peer communities were part of the PhD students’ academic lives and how these meeting arenas might impact their PhD journeys. Initial categories were: *meeting arenas for PhD students* and *community*. Meeting arenas for PhD students drew attention to arenas where the students would come together, often characterised by being informal and not structured by the university. These meeting arenas seemed to be important for the students in the establishment of peer communities. Further, the PhD students addressed the importance of community. Community referred to how they had sense of belonging to peers in the same situation as them that was an important support in their journeys.

**Table I.** The analytical process.

1. Initial analysis: peer support			
*Categories:*	Meeting arenas for PhD students	Community	
2. Dialogical analysis with AI			
*Categories:*	Community amongst PhD students	Academic development	Work and individual conditions for peer support
3. Theoretical reflection and continued analysis			
Sense of belonging (Hagerty et al., 1992; 1996)			
*PhD students’ sense of belonging and peer support*			
Informal peer arenas buffering emotional strain		Peer collaboration cultivates academic skills and professional growth	

In the second analytical round, we used an AI tool (NotebookLM) as a dialogical partner. Inspired by Bakhtin’s concept of dialogism, AI was engaged not as a neutral processor but as a dialogical partner, contributing to meaning-making through interaction with the researchers’ interpretations. This reflects Bakhtin’s view that understanding emerges through the interplay of multiple voices in dialogue (Bakhtin, 2004). We uploaded the anonymised raw material in NotebookLM, i.e., the survey and interview extracts from the theme *peer support* from the initial coding, leaving out the categories we had defined, i.e., meeting arenas for PhD students and community.

A prompt was agreed upon by the researchers and tried out before the AI analysis was conducted. The prompt was:

*In research on PhD students’ motivation in their work, I am analysing interview data from 14 informants and a qualitative survey. I want help with identifying patterns and themes in the data. Suggest categories based on the data and provide a summary of the different categories. Include a data extract that represents the different categories in a clear way.*

NotebookLM suggested the categories of *Community and social support*, *Academic development and support*, *Workplace and accommodations*, *Individual factors and challenges*, *Management and institutional support*. Because of our familiarity with the material, AI’s suggested thematic categories resonated with our initial analysis. However, we experienced that the categories were widespread and with little substance. Our analysis with NotebookLM was done through a dialogical appraoch (Bakhtin, 2004). Thus, we developed the prompt to accommodate a more thorough approach to the analytical summary from NotebookLM. The dialogical development of the prompt was:

*Can you summarise the categories into three main categories? These categories shall reflect the general tendencies in the material. Each category can have a maximum of three subpoints that summarise the categories. Include illustrative data extracts that represent the three categories in the three subpoints.*

With the revised prompt, NotebookLM suggested more substantial categories: *Community and relationships* that highlighted social support, unity, meeting places, and the importance of belonging; *Academic development and collaboration* emphasising academic exchange and support, motivation through courses and networks, and sharing experiences and guidance; *Framework conditions and individual factors* that drew attention to the importance of the workplace environment, individual needs and life phases, and fluctuation in motivation and external pressure. Consequently, we were left with detailed categories that were exemplified with data extracts.

In the third step, forming the results of this study, we returned to the material. The juxtaposition of our own analysis and using AI as a dialogical partner contributed to the development of themes. It was important, however, that we further developed the themes through a theoretical analysis with the aim of critically exploring the potential impact or consequences of the empirical material on PhD students' sense of belonging and peer communities. Also, our human reading suggested that peer support often occured in informal meeting arenas for PhD students. The attention to this detail in our reading involved a closer examination of the inital coding and the dialogical analysis done by NotebookLM. This phase involved a nearness that included reflecting with the theoretical concept of the sense of belonging (Hagerty et al., 1992, 1996). The analytical process involved approaching the categories to gain insight into the phenomenon of PhD students’ perspectives on peer support in their PhD journey, focusing on the informal meeting arenas that seemed to be important to the participants. Therefore, the three categories from the dialogical analysis with AI were reduced to two categories that illuminate peer support: *informal peer arenas buffering emotional strain*, and *belonging through academic peer collaboration*. The category of *informal peer arenas buffering emotional strain* highlight how a sense of belonging emerges in situations where PhD students come together. These encounters offer opportunities for PhD students to share challenges and successes with fellow peers. The category of *belonging through academic peer collaboration* emphasises the support offers between peers by taking part in similar academic challenges, thus leading to an increased feeling of mastering academic skills and fostering feelings of personal growth.

## PhD students’ sense of belonging and peer support

The aim of this study is to explore PhD students’ perceptions of peer support and how peers can serve as a sustaining factor throughout the PhD journey. The analysis highlights emotional and academic dimensions of peer relationships. This study indicates that informal peer arenas *buffer emotional strain* and that peer collaboration cultivates *academic skills and professional growth*, which are central to PhD students’ perceptions of peer support.

### Informal peer arenas buffering emotional strain

The following section examines how social collegial belonging and peer support serve as motivational resources for PhD students, addresses the emotional and relational dimensions of doctoral education, and explores how these factors shape students’ capacity to continue their work. This theme emphasises how PhD students establish a sense of peer community through informal social structures.

The PhD students described their relationships with peers and informal social structures as central to their motivation and well-being. Shared experiences with peers, through organised events and more spontaneous interactions, were seen as essential for navigating the challenges of their daily work.

Informal gatherings, such as shared lunches, group chats, and casual meetups, were described as inclusive and energising. These settings offered opportunities for personal connection and professional exchange, reinforcing a sense of community. These spontaneous interactions allowed students to feel intellectually and socially aligned with others in similar academic positions, for example:

*We have payday drinks sometimes, which feel inclusive and relaxed. They help to increase group cohesion and contribute to good collegial support.* (Survey participant [S])

Many of the participants considered the daily opportunities to gather, such as in shared spaces, as important in the establishment of peer relationships. For many, the significance of peer relationships was unexpected, yet surprisingly important. One participant noted that:

*It has surprised me how much it has meant... Because it was something I didn't realise I needed. I was just going to do this PhD.* (Interview participant 5 [P5])

The analysis suggests that the conditions that enable peer support are shaped by a range of contextual and individual factors, such as physical workspaces, personal circumstances, and institutional pressures. In this study, these factors appear to affect the extent and quality of peer support.

The physical environment, particularly shared office spaces, was described as both a facilitator and a potential barrier. For some, colocation created spontaneous interaction and a sense of community:

*I was initially a bit negative about the shared office, but it turned out to be really nice, because of the people around me. I think it’s good for motivation to have someone to meet and have lunch with.* (P11)

Another participant highlighted the value of shared physical spaces:

*Shared offices are of great importance for the feeling of belonging to a community.* (S)

However, other students noted that shared spaces also lead to distractions, underscoring the need for flexibility and access to quiet work environments.

*There are both pros and cons to sitting in a shared office. There’s a bit of chatting now and then. [...] You can put on a headset and... yeah. So maybe also work from home occasionally, if I feel like... now I just want to sit and write and not talk.* (P7)

These results highlight the dual role of physical space and how the work environment can serve as a critical facilitator and potential barrier. However, the colocation created spontaneous interaction and community, underscoring the human drive to belong (Baumeister & Leary, 1995). Furthermore, colocation elucidates how peer support can act as a necessary buffer against the emotional strain of doctoral work and also serves as a motivational driver to maintain engagement because of the feeling of being valued and essential to others. Hagerty et al. (1992). Accordingly, the formations of belonging seem to include forming relational bonds to other PhD students, where sharing activities and locations contribute to these formations. This sense of social connection with other PhD students, despite differences, becomes a dominant characteristic that fosters solidarity (Weiss, 1974). In the following, we further trace how peer relationships become important motivational resources for collaboration and academic development.

### Belonging through academic peer collaboration

Building on the importance of social belonging, this theme illustrates how collaborative academic engagement with peers serves to enhance learning, motivation, and a sense of mastery. Here, peer collaboration refers to how PhD students work alongside one another within individual PhD projects, leading to academic skills and professional growth. While the previous finding illustrates the importance of informal peer arenas for buffering emotional strains, the following finding illustrates how academic peer collaboration may foster a sense of belonging, both to peer communities and the academic institution.

Students described how informal discussions and collaboration served as valuable arenas for academic exchange and problem-solving during the PhD journey. These interactions created a secure environment for intellectual risk-taking and skill development, often emerging spontaneously. Yet, they played a central role in navigating research challenges. Such peer-initiated collaboration strengthened students’ sense of belonging, as they felt intellectually and socially aligned with others in similar academic positions. Such feelings of belongingness are a fundamental human motivation and a key factor in psychological well-being (Baumeister & Leary, 1995; Hagerty et al., 1996). As one student explained:

*If we have some part of a project, like if 4–5% of the project part is overlapped, we discuss, we meet all of them, this is how we can do.* (P3)

On an instrumental level, these collaborative interactions facilitated the development of concrete academic skills. Rather than relying solely on formal instruction, candidates utilised spontaneous peer contact to co-construct methodological expertise, troubleshoot coding or laboratory issues, and refine their academic writing. By engaging in low-stakes, peer-to-peer feedback cycles, students could address technical bottlenecks without the performance anxiety often associated with supervisor evaluations.

Participation in structured academic arenas, such as research schools and doctoral courses, was highlighted by the PhD students as a key source of intellectual stimulation, for example:

*I find that participating in the research school is motivating. There, I meet others who are interested in the same topics as me, make new contacts, and gain academic input in a setting that also includes enjoyable social interaction.* (S)

This illustrates how structured peer arenas foster both academic engagement and social connection, reinforcing a sense of belonging and shared purpose (cf. Strayhorn, 2012; van Kessel et al., 2025). These settings provided opportunities to engage with others working on related topics, expand professional networks, and gain new perspectives. For example, peer supported was depicted as a valuable resource for gaining insight into both academic strategies and emotional coping mechanisms:

*For me, I feel that it's really good because some PhDs who are going to finish, I can* *learn from their experience…what I should do, and how I should do it.* (P3)

This quote illustrates how professional growth and the transition from student to scholarly colleague are deeply relational processes mediated by “peer modelling.” Interacting with more advanced peers allowed PhD students to internalise the tacit norms of academic life. Seeing peers navigate the final stages of the PhD journey successfully provided a realistic blueprint for their own progression, normalising setbacks and demystifying the “hidden curriculum” of academia. Consequently, this collective socialisation helped mitigate the imposter syndrome and fostered a resilient professional identity.

Crucially, these dynamics were underpinned by abundant informal learning opportunities that occurred outside formal coursework. Spontaneous corridor discussions, lunch-table brainstorming sessions, and shared office dialogues served as unstructured, safe spaces for cognitive and emotional development. In these environments, PhD students felt free to ask fundamental questions and share vulnerabilities, translating everyday social interactions into valuable catalysts for both skill mastery and institutional belonging.

Intertwined with the sense of belonging to collegial peer communities, individual needs and life circumstances also shape how students connect to communities. Factors such as personality, caregiving responsibilities, and various energy levels influenced participation in social and academic activities:

*I’m not the one who participates the most because I have small children, but the atmosphere is always pleasant, and there’s never any conflict.* (S)

Although there is a recognition of the social gatherings as important, the example illustrates that a PhD life consists of balancing between home-obligations and social bonding.

These insights show that, although peer support is valued, it cannot be taken for granted. Its impact depends on whether peer relationships meet students’ psychological needs for connection and affirmation, particularly during periods of low motivation or high stress (Baumeister & Leary, 1995; Gonzalez et al., 2021). It is also shaped by the interplay between institutional structures, physical environments, and individual circumstances. The findings suggest that PhD students’ sense of belonging is closely tied to informal social interactions, where working alongside peers may itself function as a form of support through shared activities and learning.

## Discussion

This study highlights the multifaceted role of peer support in doctoral studies, revealing it as a resource that complements formal supervision and has the potential to enhance both academic and emotional well-being. Drawing on the theoretical framework of sense of belonging (Baumeister & Leary, 1995; Hagerty et al., 1992, 1996), the results suggest that peer support strengthens PhD students’ experience of being connected to their academic environment and has the potential to contribute to PhD students’ motivation. A sense of belonging thus appears to be essential for motivation, persistence, and mental health.

The findings show that peer support functions as a relational infrastructure helping students manage the emotional and academic demands of doctoral work. Through informal, reciprocal interactions, PhD students build trust and shared purpose, even in solitary research settings. This demonstrates that peer support among PhD students fosters a sense of collegial belonging, which serves as a motivational resource. Thereby, support from peers can be considered to address the potential variation in motivation often registered among PhD students (Gonzalez et al., 2021).

This study shows how peer support is enacted in everyday doctoral life, not only through structured programmes but also through spontaneous, student-led initiatives. Accordingly, these informal arenas are crucial for building belonging and sustaining motivation amongst PhD students. In the context of doctoral education, peer relationships can be understood as a response to the fundamental human motivation to belong (Baumeister & Leary, 1995). In this study, informal social structures, such as shared lunches, group chats, and casual meetups, were described as energising and inclusive, helping to reduce feelings of isolation and reinforcing a sense of shared purpose. Thereby, the participants described peer support as a buffer against the emotional strain of doctoral work and the potential feeling of isolation from a collegial community.

The concept of ‘common-concern relationships’ (Weiss, 1974) is relevant here, as students bond over shared challenges and aspirations, creating a sense of solidarity that transcends disciplinary boundaries. Although the participants differed in many respects, their shared position as PhD students was a dominant aspect of their lives, making social connections with other PhD students crucial for fostering a sense of belonging.

By systematically anchoring adjacent concepts, such as motivation, collaboration, and informal learning, within Hagerty et al. (1996) dual-dimension model, we can clarify pathways through which peer support operates. We argue that informal social spaces primarily cultivate the dimension of valued involvement. When PhD students participate in non-evaluative social structures (e.g., lunches, chats, payday drinks), they experience feeling personally accepted, valued, and emotionally secure. This valued involvement acts as an emotional buffer against the psychological strain of doctoral work, directly sustaining their intrinsic motivation.

Conversely, academic peer collaboration primarily drives the dimension of fit (or alignment) (Hagerty et al., 1992, 1996). When doctoral candidates engage in joint problem-solving, project discussions, and peer modelling, they align their personal research goals, academic standards, and professional values with those of the wider scholarly community. This dual alignment of valued involvement (social-emotional) and fit (intellectual-professional) is what ultimately prevents conceptual fragmentation in the student experience, establishing a holistic, resilient foundation for academic belonging.

To support students' academic and social development, institutions should recognise the importance of informal peer spaces and facilitate opportunities for peer engagement, collaboration, and community-building (Martínez-García et al., 2024, 2025). When students feel emotionally supported by their peers, as suggested by the participants, they are more likely to perceive academic challenges as manageable, which in turn can affect their engagement and resilience. These results align with previous research, showing that peers deem it essential to support one another, offering emotional and academic benefits (Boud & Lee, 2005; Gardner, 2010; Wegener et al., 2016). This study adds to current knowledge on peer support by emphasising the informal meeting arenas. Student-initiated social gatherings contributed to the establishment of peer relationships and, consequently, arenas where they might experience peer support. This study provides important insight into how collegial belonging is connected with informal peer support, which may serve as a motivational resource for the individual's PhD work.

Building on the importance of social belonging, the study illustrates how peer collaboration plays a central role in fostering academic development and professional identity. Facilitating peer interaction and collaboration allows institutions to enhance these benefits (Martínez-García et al., 2024, 2025). This study shows that peer collaboration is connected with opportunities for academic growth through social interactions and more experienced peers serving as models for others.

The participants in this study expressed that peer students contributed to overcoming complex research challenges in safe settings. Research supports that a sense of belonging can be connected to academic success, student retention and satisfaction (Strayhorn, 2012; van Kessel et al., 2025), and that meaningful social interactions are key to fostering belonging among students (Amin et al., 2025). Through shared learning and reciprocal feedback, students gain opportunities to develop mastery experiences that strengthen their confidence in their ability to manage future challenges. Meschitti (2019) similarly found that peer learning environments, such as informal discussions and mutual feedback, foster intellectual risk-taking and skill development. Research mirrors the results in this study, highlighting how informal and formal peer meetings, as a supplement to supervision, can contribute to research skill development (Wang and Min, 2023). Interactions with peers further offered relatable models for success and help normalise the ups and downs of doctoral work. This is especially relevant in light of Fangqi and Pengpeng (2024) results, which show that lack of affirmation can lead students to question their academic value. Peer modelling thus plays a corrective role by reinforcing belief in one’s potential.

This process of academic growth and professional identity socialisation through peer collaboration is theoretically elucidated by Lave and Wenger (1991) CoP framework. Within this constructivist learning paradigm, the doctoral trajectory is conceptualised not merely as the individual acquisition of cognitive knowledge, but as a journey of situated social learning. Junior PhD students enter the academic community as “newcomers” engaging in what Lave and Wenger (1991) call “legitimate peripheral participation”.

By collaborating on authentic academic tasks, such as peer-review groups, writing workshops, and joint problem-solving, PhD students co-construct essential academic skills while dynamically interacting with more experienced peers. This relational practice allows students to gradually move from peripheral to central, legitimate positions within their disciplinary community. Through this socially situated transition, PhD students do not just acquire “skills” in isolation, they experience a change of their scholarly identity and research self-efficacy, actively embodying the values and practices of the academic collective (Boud & Lee, 2005; Gonzalez et al., 2021).

The motivational impact of structured academic peer arenas, such as research schools, illustrates the importance of institutional support in enabling peer collaboration (Martínez-García et al., 2024, 2025) and can be experienced as a positive academic contribution for the participants in this study. These settings combine academic stimulation with social interaction, creating spaces where PhD students can both learn and belong. Participants in this study emphasised that both formal arenas, such as research schools, and informal ones, such as lunches, strengthen their sense of peer support while offering opportunities for academic development. This development relates not only to their individual projects, but also to the process of becoming part of academia.

## Limitations

Several limitations should be acknowledged when interpreting the findings of this study. First, the study’s empirical basis is limited to participants recruited from a single Norwegian university. Because doctoral education in Norway is characterised by a distinctive employment-based model where PhD students are hired as university staff with financial stability and professional benefits, the findings may not be directly transferable to international contexts where PhD studies are often self- or scholarship-funded. Second, participation in both the qualitative survey and the semi-structured interviews was entirely voluntary, which introduces a potential self-selection bias. It is possible that PhD students who already held strong views on, or had active experiences with, peer support and academic belonging were more inclined to participate.

Furthermore, the researchers' insider positions as current or recently graduated PhD students must be considered. While our shared experiences with the participants enhanced trust and facilitated deep, reflective dialogues during interviews, this proximity to the field may also have influenced our assumptions and interpretations during the analytical process. To ensure ethical and analytical awareness, we continuously acknowledged the participants as true experts on their own lives.

Finally, the integration of the AI tool NotebookLM as a dialogical partner in the thematic analysis introduces a unique methodological nuance. While this dialogical approach challenged our initial interpretations and helped identify nuanced patterns across the survey and interview data, it also carries risks of algorithmic influence. We mitigated this by maintaining a rigorous familiarity with the raw dataset, manually checking and validating all AI-generated categories and illustrative quotes to ensure the final analysis remained strictly researcher-led and grounded in the participants' actual voices.

## Implications

This study offers important implications for doctoral education. The study advances the literature by demonstrating that a ‘sense of belonging’ in doctoral journeys is not a monolithic experience, but rather enacted through informal, student-led initiatives that complement formal structures. By applying Hagerty et al. (1996) framework, we show that informal social peer support and academic peer collaboration are not interchangeable; rather, they serve complementary functions. Informal interactions primarily secure valued involvement (emotional safety and mutual recognition), while collaborative practice-based learning secures fit (professional and intellectual alignment). This distinction helps prevent the conceptual fragmentation often seen in doctoral well-being research. This frame belongs as a dynamic, relational process where reciprocal peer interactions directly strengthen engagement and resilience.

Processes of informal peer support are deeply shaped by institutional and individual contexts, particularly within employment-based models like the Norwegian higher education framework. In this context, where PhD students navigate a dual status as both learners and salaried university employees, informal peer communities serve as a vital, yet under-researched, buffer. Such everyday, low-stakes interactions allow students to transition from peripheral newcomers to central, legitimate contributors Lave and Wenger (1991).

This study points to a broader institutional responsibility. Peer support should be recognised not as a discretionary supplement to supervision, but as a central component of doctoral education. This requires institutions to play a key role by recognising informal peer spaces and facilitating and safeguarding student-initiated “relational infrastructures” such as shared lunches and spontaneous corridor dialogues. Ultimately, supporting students’ well-being, persistence, and completion demands a systemic shift in focus from individual performance to collective well-being, highlighting the importance of cultivating inclusive and flexible peer dynamics across doctoral programmes.

## Closing remarks

While supervision remains central to doctoral education, this study demonstrates that peer support plays a distinct and indispensable role in fostering academic motivation. The study shows that peer relationships have the potential to contribute to a sense of belonging, which is essential for sustaining motivation throughout a PhD journey.

Peer support was widely valued by the participants, but its accessibility was shaped by contextual and individual factors. Shared office spaces were described as both enabling and distracting, depending on social dynamics and individual preferences for interaction or solitude. Factors such as caregiving responsibilities and personality traits further influenced participation in peer activities, underscoring the need for flexible and inclusive peer environments.

This study highlights the institutional responsibility to enable inclusive and responsive peer support structures. Beyond pedagogical implications, the findings also carry political significance. Informal peer support reflects relational labour that often compensates for gaps in formal structures. Such networks foster both academic engagement and emotional safety, underscoring the need for institutions to invest in environments that support collective care and belonging.

## Data Availability

The dataset is in Norwegian and is not publicly available to protect the anonymity of participants. Data may be available from the corresponding author if a reasonable request is made. The integrity and anonymity of the participants will also be maintained in this case.
